# The case for gender considerate tobacco control policies in Albania

**DOI:** 10.1186/s41256-020-00143-6

**Published:** 2020-04-14

**Authors:** Harminder Guliani, Monika Çule

**Affiliations:** Department of Economics, University of Regina, 3737 Wascana Parkway, Regina, SK Canada

**Keywords:** Albania, Tobacco control, Gender-based smoking behavior, Multilevel analysis, Two-stage residual inclusion

## Abstract

**Background:**

Tobacco use is a serious health concern in Albania. While the prevalence of tobacco smoking has traditionally been higher for men, the increasing prevalence of smoking for women is becoming a concern. The 2007 Tobacco Control policy mandated smoke-free indoor spaces, banned various forms of tobacco advertising, required written health warnings on packaging and levied excise taxes on cigarette sales. Since smoking behavior varies among different demographic groups, each group’s response to a uniform policy will differ, blunting the effectiveness of these efforts as a result. This study examines various socioeconomic, demographic and behavioral factors affecting both the likelihood and frequency of smoking in Albanian households in order to provide insights on targeting various populations more effectively.

**Methods:**

The study utilizes data from Albanian 2008–09 and 2017–18 Demographic and Health Surveys consisting of adults aged 15–49 years. The outcome variable includes respondents’ current tobacco smoking behaviour and its frequency. The exposure variables include respondents’ sociodemographic and lifestyle characteristics. We use a two-level random intercept model with the two-stage residual inclusion estimation method to determine the association between outcome and exposure variables. By including a time variable, we capture the change in smoking behavior during the 2009–2018 period. We also extend the analysis by assessing the differential influence of gender on the likelihood of smoking, both by income quintiles and education.

**Results:**

The results suggest that the likelihood of smoking decreased by 23% in 2017–18 compared to 2008–09, after controlling for various socioeconomic and demographic factors. Tobacco smoking is also found to be linked to alcohol consumption, with binge drinkers 59% more likely to smoke tobacco compared to moderate drinkers. We also found significant inter-quintile and inter-educational differences in smoking practices within each gender category. While the likelihood of tobacco smoking decreases with increasing wealth and educational attainment among men, the opposite (for wealth) or more involved (for educational attainment) patterns are true among women.

**Conclusions:**

To further enhance the effectiveness of the current Tobacco Control policy, the Government of Albania should target various demographic groups (such as poor males, rich and educated females) in a differentiated fashion.

## Background

Tobacco remains an important public health issue in the European region as its use is responsible for 16% of all deaths in adults over 30 [1]. Within the European region, tobacco smoking is the highest in the post-communist Eastern European countries. The World Health Organisation (WHO) (2018) data on the age-standardized prevalence of tobacco smoking among 15 years and older shows that in 2016, most countries in Eastern Europe have smoking rates well above the European regional average of 28.7% [2]. However, the prevalence of smoking varies considerably within the Eastern European nations, ranging from 29.2% in Albania to 46% in Montenegro [2]. While in some countries such as Bulgaria and the Czech Republic the smoking prevalence is very high among both males and females, in other post-communist countries female smoking rate is considerably lower than males, but it is increasing [3]. As a result, the gap in smoking prevalence between male and female adults is narrowing down for many of these transitional countries. The fall of communism, privatisation of state-owned tobacco monopolies with aggressive tobacco marketing policies, and the increasing social acceptability among females are among the contributing factors [3–5].

In Albania, tobacco use is becoming a serious health concern [6]. The transition to a market-based economy in the early 1990s resulted in a significant increase in the availability of imported tobacco products. At the same time, smoking among young females became more socially acceptable [5]. In the absence of any tobacco control regulation, the prevalence of smoking increased considerably [7]. According to various estimates, the rate of tobacco smoking for Albanian men is more than 40% [5, 7–9]. Although more recently, the Albanian Demographic and Health Survey (ADHS) reported a decrease to 36% [10]. While the prevalence of tobacco smoking among females is much lower at 5% (in 2018), it has been rising [5, 10]. Comparatively, these smoking rates are higher than the averages in high-Human Development Index countries [9]. Overall, the premature death toll attributable to smoking is estimated at 50% (399,000 out of 797,840) of the smoking population [11]. With tobacco smoking responsible for 4110 deaths annually, the economic costs due to direct health expenditures and loss in productivity total 34.68B ALL (appx USD 315 M) [9].

In response to the smoking epidemic, the Albanian government signed the WHO Framework Convention on Tobacco Control (FCTC) in 2004 and ratified it in 2006 [6]. The inaugural legislation, Law No. 9636 on Health Protection from Tobacco Products was enacted in November 2006 and was amended in 2008, 2011, 2013 and 2014 [12]. Consistent with WHO FCTC, these policies mandated smoke-free indoor public spaces, banned various forms of tobacco advertising, required written health warnings on cigarette packages and levied excise taxes on cigarette sales [6, 11]. If fully implemented in line with WHO FCTC, this multipronged policy approach is projected to reduce smoking prevalence by 44% within 15 years [11]. The WHO (2019) report on the global tobacco epidemic which, provides snapshot assessments for various aspects of the tobacco policy, show little to no change in the tobacco control efforts in Albania since 2016. The slight increase in the excise tax on cigarettes from 45 to 49% was the exception [6, 13]. Despite the progress, there is considerable scope to further develop, implement and enforce the current policy.

It is important to note, however, that smoking behavior varies among different demographic groups. Notably, the smoking behavior of females and males differ due to varying gender social norms and motivations to start or quit smoking, as well as different health risk factors [14]. As a result, their response to uniform policy efforts may differ as it is reflected in the differential change in the smoking prevalence rates for men and women. More specifically, under the current tobacco control policy in Albania, while the smoking rate for men declined from 43% in 2009 to 36% in 2018, it increased for women from 4 to 5% [10]. Therefore, to effectively address the smoking behavior of different demographic groups, there is a need for designing and delivering programs that are informed by a proper understanding of factors influencing the smoking behavior in a gender context.

There are a limited number of studies examining tobacco smoking among adult and youth populations in Albania. Ross at al. (2008) provides a statistical account of smoking prevalence among various demographics utilizing the results of the 2007 Albanian Adult Tobacco Survey (AATS1) [7]. In addition to AATS1, Zaloshnja et al. (2010) utilized the 2009 survey (AATS2) and found a statistically significant increase in smoking prevalence across all demographics, with the highest increase among females [5]. The study concluded that due to lax enforcement, the newly implemented policy had not yielded any deterring effect in smoking behavior and its prevalence.

A few other studies focus particularly on risky health behaviors (including smoking) among Albanian youth [15–17]. Pipero et al. (2015) for instance examined the socio-economic correlates of risky health behaviors (smoking, alcohol drinking and BMI) of youth aged 15–24 using data from 2008 to 2009 ADHS [15]. Using data from the 2011 wave of European School Survey Project on Alcohol and other Drugs (ESPAD), Toçi et al. (2014) examined the social and demographic factors of the lifetime prevalence of smoking, alcohol drinking and use of cannabis among 15–16-year students [16]. In addition, utilizing data of both 2011 and 2015 waves of ESPAD, Toçi et al. (2017) focused particularly on smoking behavior and found that cigarette smoking prevalence declined in 2015, suggesting that the tobacco control measures played a role in smoking deterrence [17].

Despite utilizing different survey data and methodology, a common finding of these studies is that smoking prevalence among males is much higher than among females. This finding, however, does not negate the concerning observation that, although lower, smoking prevalence among females is on the rise and special attention is needed to contain the problem. As mentioned earlier, this pattern of smoking prevalence is not unique to Albania and concerns several other post-communist countries.

Given this context, using the 2008–09 and 2017–18 ADHS data, our study examines various socioeconomic, demographic and behavioral factors affecting the likelihood and frequency of smoking behavior in Albanian households with the objective of answering three questions. First, what factors affect smoking behaviors in Albania? Second, do income and education related gender differences affect the likelihood of smoking? If demographic differences are found to affect the likelihood of smoking, then our findings would be very relevant to the development of more effective tobacco controls. To the best of our knowledge, this is the first nationwide study assessing the differential influence of gender on the likelihood of smoking, both by income quintiles and education. WHO FCTC has continuously emphasized the importance of implementing gender-specific tobacco control strategies [3, 4, 14] and the policy implications that follow from our analysis make a valuable policy contribution in this regard.

Third, we examine whether the Tobacco Control policy of 2007 has deterred smoking behavior during the 2009–2018 period. While Zaloshnja et al. (2010) find no immediate impact of the 2007 Tobacco Control policy [5], Toçi et al. (2017) suggest that tobacco control measures played a role in the decline of smoking prevalence among youth in 2015 compared to 2011 [17]. Given the non-experimental nature of our data, we are unable to assess the effectiveness of the 2007 Tobacco Control policy. However, by including a time variable in the econometric analysis, we capture the change in smoking behavior over the period 2009–2018. Given the ADHS larger sample size and the comprehensive coverage of age groups, our analysis sheds some light on whether the Tobacco Control policy of 2007 has yielded any results during the 2009–2018 period.

## Methods

### Study design and data source

The analysis uses the 2008–09 and 2017–18 ADHS data conducted by the Institute of Public Health and the Institute of Statistics with technical assistance from ICF International and funded by the international agencies. The DHS is a large-scale, cross-sectional household survey that uses a multistage cluster sample design to collect information on nationally representative samples of males and females. ADHS samples were selected using a stratified, two-stage cluster sampling design.^1^ The survey’s primary objective is collecting information, at the national and regional level, on respondents’ various socio-economic and demographic characteristics such as age, education, wealth index, place of residence, employment status, as well as data on health related lifestyle such as smoking tobacco. The overall sample in this study consists of 25,986 adults between the ages of 15–49 years,^2^ 18,444 females and 7542 males. The 2008–09 data consists of 10,597 adults, 7584 females and 3013 males. The 2017–18 data consists of 15,389 adults, 10,860 females and 4529 males.

### Study variables

#### Outcome variables

“Current tobacco smoking” – the outcome variable – is based on responses provided to the questions if the respondent currently smokes cigarettes or any other type of tobacco such as cigars, pipes, cheroots or cigarillos during the survey years.^3^ If the respondent (aged 15–49) did not smoke any tobacco products, a binary dependent variable is created with a value of zero (0). If the respondent indicated smoking one or more types of tobacco, the variable takes the value of one (1).

Consistent with existing studies [7], the outcome variable - frequency of smoking - is measured by the number of cigarettes smoked in the last 24 h conditional on smoking cigarettes. Alternatively, the frequency of smoking could relate to how often the person smokes (e.g. every 5 min; every 30 min or every hour), but such information is not available in our data set.

#### Exposure variables

Consistent with the existing literature, the independent variables include respondent’s sociodemographic and lifestyle characteristics such as respondent’s gender, age, educational attainment, marital status, survey year (2008–09, 2017–18), occupation, health awareness, and alcohol consumption. The survey year dummy allows us to observe any possible differences in tobacco smoking at different times. The awareness variable indicates the respondent’s perception of health problems from smoking. A binary variable “health awareness on smoking” is created with a value of one (1) if the respondent believes that smoking causes serious to minor health problems and zero (0) otherwise.^4^

The prevalence of alcohol consumption is based on responses on consumed alcohol such as beer, wine, raki, or other spirits in the last 12 months and the average number of drinks on the drinking day. The variable is divided into three categories: “non-drinkers”, for those who reported not consuming alcohol; “moderate drinkers” for those who reported consuming less than 5 (or 4 for females) on a drinking day and “binge drinkers” for those who reported consuming 5 or more drinks (4 or more for females).

Other independent variables at household-level include religion and household economic status, measured by wealth index.^5^ Each household is classified into quintiles where the first quintile is the poorest 20% of households and the fifth quintile is the wealthiest 20% [18]. At the community-level, the place of residence (urban/rural), and the administrative prefectures/regions capture the differences in the availability and accessibility of tobacco products between urban and rural areas and among different prefectures. Table 1 provides summary statistics for the dependent and independent variables.

**Table 1:** Summary Statistics for the dependent and independent variables (*N* = 25,986)

Variables	Mean	Standard Deviation
***Dependent Variable***		
Smoking Tobacco	0.219	0.414
Frequency of Smoking	18.318	0.791
***Individual-level independent variables***		
Gender		
Male	0.502	0.500
2017–18 Survey Year	0.468	0.499
Age		
15–19	0.185	0.388
20–24	0.142	0.349
25–29	0.122	0.327
30–34	0.113	0.317
35–39	0.130	0.336
40–44	0.149	0.356
45–49	0.158	0.365
Marital Status		
Not Married	0.385	0.487
Married	0.595	0.491
Divorced	0.020	0.141
Education		
No Education/Primary less than 4- year	0.019	0.136
Primary 8 year	0.400	0.490
Secondary/Professional/Technical	0.405	0.491
University and Post-graduate	0.177	0.381
Occupation		
Unemployed	0.459	0.498
Professional/Clerical/Sales/Services	0.215	0.411
Agriculture	0.110	0.313
Unskilled Manual/Other	0.061	0.238
Skilled Manual	0.155	0.362
Alcohol consumption		
Non drinker	0.546	0.498
Moderate Drinker	0.435	0.496
Binge drinker	0.011	0.103
Health awareness on Smoking	0.981	0.137
***Household-level independent variables***		
Household wealth quintile		
Quintile 1 (Very Poor)	0.185	0.388
Quintile 2	0.198	0.398
Quintile 3	0.203	0.402
Quintile 4	0.202	0.402
Quintile 5 (Very Rich)	0.212	0.409
Religion		
Orthodox	0.076	0.265
Catholic	0.107	0.309
Bektashi	0.015	0.123
Islam	0.784	0.412
Atheist	0.013	0.114
Other	0.005	0.072
***Community-level independent variables***		
Place of residence		
Urban	0.521	0.500
Administrative regions		
Berat	0.047	0.211
Dibër	0.049	0.216
Durrës.	0.085	0.279
Elbasan	0.114	0.318
Fier.	0.104	0.305
Gjirokastër.	0.026	0.158
Korçë	0.075	0.263
Kukës	0.030	0.170
Lezhë	0.042	0.200
Shkodër.	0.095	0.294
Tiranë	0.282	0.450
Vlorë	0.052	0.222

### Data analysis

Many unobserved characteristics of the community including peer influences, social norms, and smoking culture can, in part, influence an individual’s decision to smoke tobacco. Therefore, the probability of smoking is likely to be correlated among community members. This leads to biases in the application of standard logistic regression models [19]. Therefore, we use a two-level (individual–household and community) random intercept logistic model which also corrects standard errors of the estimated coefficients for intra-cluster correlation (heteroscedasticity).

Since smoking tobacco and drinking alcohol often occur together, we included alcohol consumption as an explanatory variable. While those consuming alcohol are more likely to smoke tobacco, causation may operate in the reverse direction. Moreover, some unobserved characteristics may influence both alcohol and tobacco consumption, making alcohol consumption endogenous and hence producing an upward bias in its estimates. In addition to multilevel modeling, following Terza et al., (2008) recommendation on addressing endogeneity in empirical research in health economics, we use the two-stage residual inclusion (2SRI) estimation method [20]. The first stage equation of the 2SRI specifies drinking alcohol (those who drinks 1–5 days per week versus those who drink 1–3 days per month or less) as a function of exogenous variables and the second-stage of the 2SRI approach estimate the likelihood of smoking tobacco and its frequency by including the residual computed from first stage estimation as a regressor. A negative binomial count data model is used to analyze factors affecting the intensity of smoking.^6^ Standard weights from the men’s and women’s files are used to adjust for the unequal probability of selection. These weights are first de-normalized [21]. STATA version 15 was used for all data analyses.

## Results

### Descriptive analysis

Table 2 reports tobacco smoking (in percentages) by selected socioeconomic and demographic characteristics.

**Table 2:** Weighted Prevalence of Tobacco smoking by Selected Variables (%)

	Pooled data ***N*** = 25,986	2008–09 ***N*** = 10,597	2017–18 ***N*** = 15,389
**Average**	21.94	23.12	20.60
**Household Income quintile**			
Very Poor	19.67	20.11	19.21
Poor	20.24	22.20	18.02
Middle quintile	21.28	22.12	20.23
Rich	23.76	25.16	22.18
Very Rich	24.42	25.59	23.09
**Sex**			
Male	39.21	42.52	35.57
Female	4.54	4.18	4.97
**Education**			
No education/Primary less than 4-year	33.67	33.79	33.56
Primary 8-year	21.98	22.14	21.76
Secondary/Professional/Technical	21.59	22.73	20.21
University and Post-graduate	21.41	26.38	18.37
**Alcohol Consumption**			
Non drinker	10.18	10.26	10.01
Moderate Drinker	35.75	37.87	33.16
Binge Drinker	54.42	59.34	48.10
**Place of residence**			
Urban	23.86	25.23	22.67
Rural	19.86	21.38	17.51
**Administrative Regions**			
Berat	19.57	17.85	22.38
Dibër	20.09	23.94	14.99
Durrës.	21.02	26.21	15.82
Elbasan	23.23	27.04	17.58
Fier	18.07	14.82	22.09
Gjirokastër.	20.98	20.23	22.14
Korçë	19.08	24.70	13.92
Kukës	17.86	22.39	12.85
Lezhë	21.24	26.94	14.94
Shkodër.	16.79	15.77	18.63
Tiranë	25.81	26.10	25.56
Vlorë	28.26	30.14	26.17

**Fig. 1:**
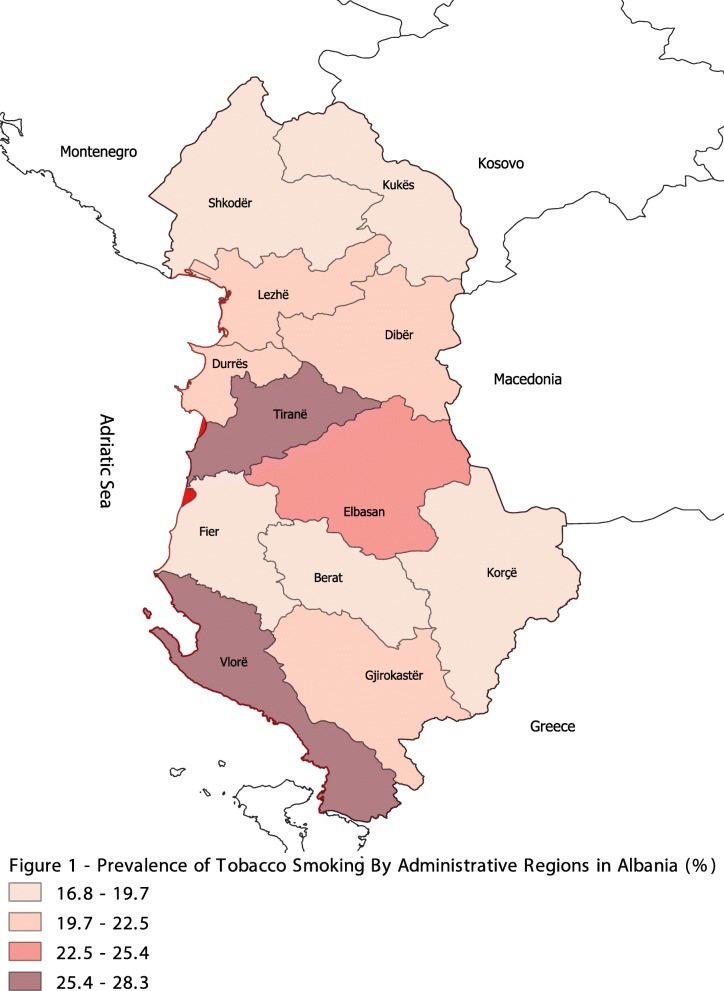
Prevalence of Tobacco Smoking By Administrative Regions in Albania (%)

Overall, the weighted prevalence rate of tobacco smoking was 21.94%. However, wide variations are noticeable by the respondent’s economic status, education, alcohol consumption, residence, and administrative regions. On average the prevalence rate declined slightly between the two survey years, from 23 to 21%. This decline is consistent across various socioeconomic and demographic characteristics with the exception of females and certain regions. While the smoking rate declined for males from 43 to 36%, it increased slightly for women from 4 to 5%. Figure 1 shows the prevalence rate of tobacco smoking by administrative regions in Albania from the pooled data. The prevalence rate in Vlorë, Tiranë, and Elbasan regions was found to be above the national average. However, between the two survey years, the smoking rate increased in Berat, Fier, Gjirokastër, and Shkodër as shown in Table 2.

**Fig. 2:**
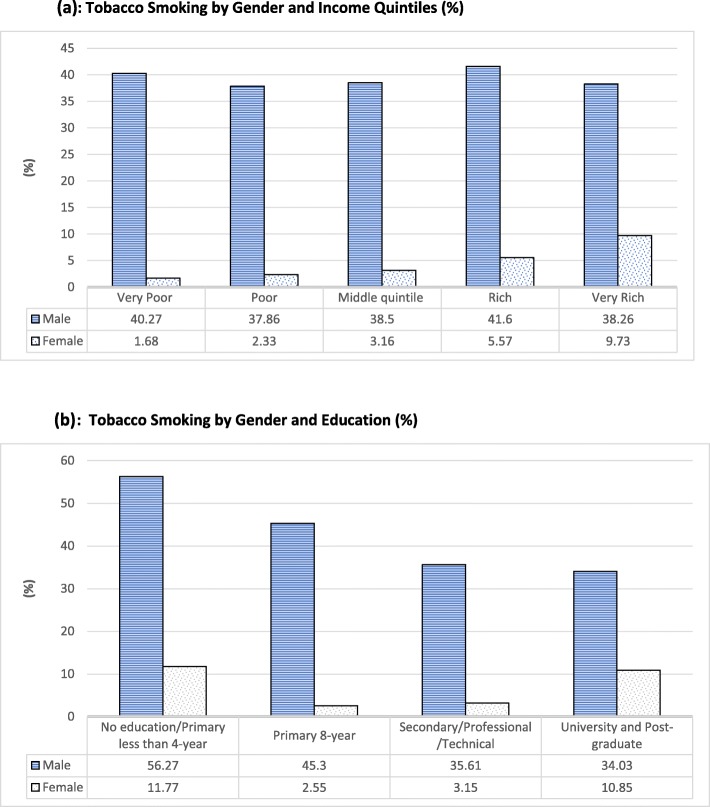
**a** Tobacco Smoking by Gender and Income quintiles (%). **b** Tobacco Smoking by Gender and Education (%)

Figure 2(a) shows the variation in tobacco smoking by gender and income quintiles (pooled data). While poor males are more likely to smoke than rich males, rich females are (almost six times) more likely to smoke than poor females. Fig. 2(b) illustrates the variation in tobacco smoking by gender and education. While tobacco-smoking decreased for males with increased education, women with higher education are more likely to smoke tobacco than those with secondary education.

**Fig. 3:**
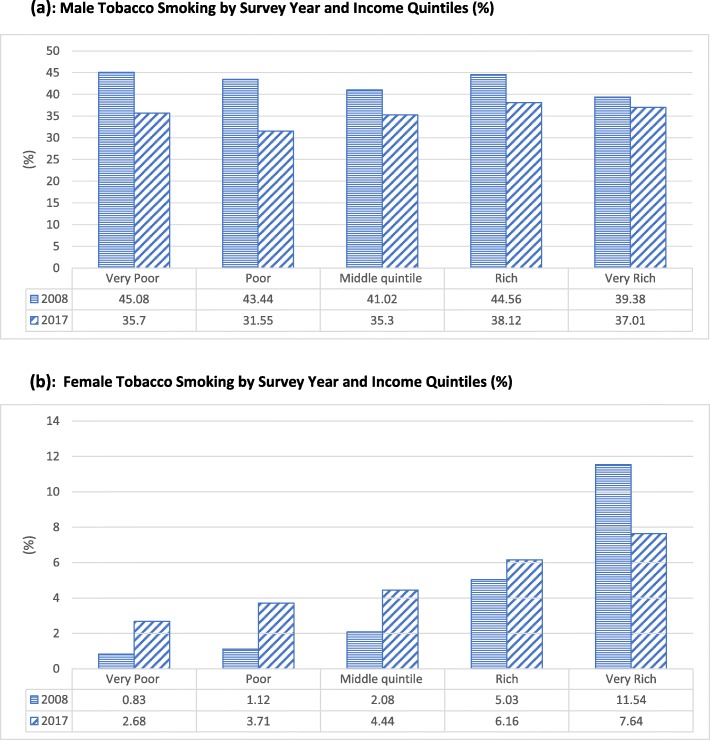
**a** Male Tobacco Smoking by Survey year and Income quintiles (%). **b** Female Tobacco Smoking by Survey year and Income quintiles (%)

Figure 3(a) and (b) show variation in male and female tobacco smoking by survey year and income quintiles. The figures reveal an interesting divergence of tendencies, in the prevalence of smoking, between the genders. Specifically, while tobacco smoking over the two survey years decreased within each quintile among males, the prevalence of smoking increased within each quintile among females, except for very rich females. However, this decrease among very rich females, over the two survey years, is not statistically significant at the 5% level. Although in 2017–18 survey year very rich females smoke less compared to 2008–09 survey year, the gradient persists in both years indicating that very rich females are more likely to smoke than poor females in both survey years.

**Fig. 4:**
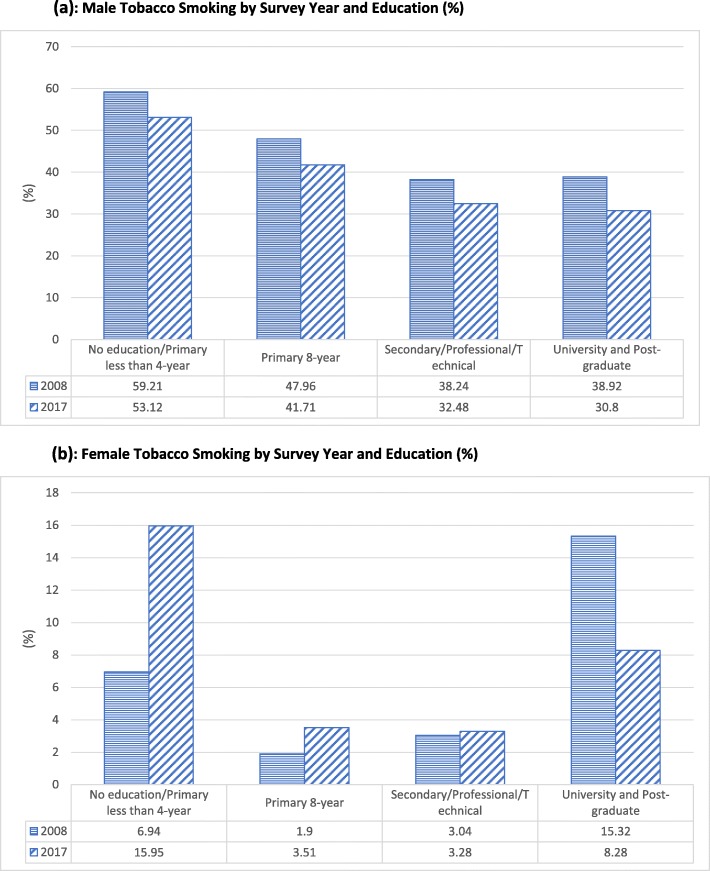
**a** Male Tobacco Smoking by Survey year and Education (%). **b** Female Tobacco Smoking by Survey year and Education (%)

Figure 4(a) and (b) show variation in male and female tobacco smoking by survey year and education. The first important observation is that consistent with the pooled data, tobacco-smoking decreased for males with increased education in both 2008–09 and 2017–18 survey years. Similarly, consistent with the pooled data, women with higher education are more likely to smoke tobacco than those with secondary or primary education in both survey years. The second important observation is that for males, tobacco smoking decreased over the two survey years within each education level. Conversely, for females, the prevalence of tobacco smoking, between 2009 and 2018, increased among all education levels, except among university and post-graduate females. Although the smoking prevalence, between the two survey years, increased among the uneducated and those with less than 4 years of primary education, the difference is not statistically significant. Similarly, the decrease in smoking prevalence among university and post-graduate females, between the two survey years, is not statistically significant.^7^

On average, respondents reported to smoke 18 cigarettes in the last 24 h preceding the survey. However, the mean number of cigarette smoking is lower among females, very rich, and respondents with university education.

### Econometrics results

Table 3 reports the regression results for the likelihood of smoking.^8^ The likelihood-ratio test favors the random-intercept logistic over the ordinary logistic model. To better understand the female smoking behavior, we assess the differential influence of gender on the likelihood of smoking, both by income quintiles and education, by extending the regression model with two sets of interaction terms. More specifically, the interaction terms in Model 2 allow comparing inter-quintile differences in smoking practices within each gender category while using poor and very poor (bottom two quintiles) as the reference category. The interaction terms under Model 3 allow comparing inter-educational differences in the likelihood of smoking within each gender category, using university education as the reference category.

**Table 3:** Multilevel Logistic Regression results for Smoking Tobacco

	Model 1	Model 2	Model 3
**Variables**	**Odds ratio** **(95% CI)**	**Odds ratio** **(95% CI)**	**Odds ratio** **(95% CI)**
***Fixed part***			
***Individual-level variables***			
Survey year (ref: 2008–09)	0.767* (0.693, 0.853)	0.760* (0.685, 0.843)	0.745* (0.671, 0.827)
Gender (ref: Male)	13.161* (11.759, 14.731)	25.937* (21.140, 31.822)	5.236* (4.274, 6.414)
Age (ref: 15–19)			
20–24	3.150* (2.561, 3.874)	3.253* (2.645, 4.001)	3.352* (2.726, 4.123)
25–44	4.217* (3.436, 5.175)	4.308* (3.510, 5.286)	4.586* (3.734, 5.634)
45–49	3.397* (2.687, 4.295)	3.461* (2.737, 4.376)	3.688* (2.913, 4.668)
Education (ref: University and Post-graduate)			
No education/Primary less than 4-year	2.459* (1.756, 3.445)	2.694* (1.906, 3.808)	–
Primary 8-year	1.352* (1.152, 1.587)	1.431* (1.221, 1.676)	–
Secondary/Professional/technical	1.075 (0.931, 1.240)	1.104 (0.959, 1.270)	–
Gender X Education			
Male X No education/Primary less than 4-year	–	–	3.017* (1.963, 4.636)
Male X Primary 8-year	–	–	2.392* (1.976, 2.895)
Male X Secondary/Professional/technical	–	–	1.653* (1.387, 1.969)
Female X No education/Primary less than 4-year	–	–	1.746** (1.100, 2.771)
Female X Primary 8-year	–	–	0.515* (0.409, 0.650)
Female X Secondary/Professional/technical	–	–	0.603* (0.487, 0.747)
Alcohol consumption (ref: Moderate Drinker)			
Non drinker	0.421* (0.378, 0.468)	0.435* (0.391, 0.483)	0.438* (0.394, 0.487)
Binge drinker	1.589** (1.101, 2.293)	1.526** (1.057, 2.202)	1.529** (1.058, 2.209)
Health awareness on Smoking	0.542* (0.395, 0.743)	0.528* (0.382, 0.730)	0.537* (0.390, 0.740)
Marital Status (ref: Not married)			
Married	0.923 (0.810, 1.056)	0.927 (0.812 1.057)	0.932 (0.817, 1.062)
Divorced	2.678* (2.021, 3.574)	2.711* (2.032, 3.616)	2.751* (2.063, 3.667)
Occupation (ref: Unemployed)			
Professional/Clerical/Sales/Services	1.437* (1.254, 1.646)	1.389* (1.214, 1.589)	1.334* (1.165, 1.528)
Skilled Manual	1.607* (1. 398, 1.849)	1.541* (1.340, 1.771)	1.452* (1.259, 1.675)
Agriculture	1.077 (0.904, 1.282)	1.031 (0.862, 1.233)	0.987 (0.825, 1.181)
Unskilled Manual/Other	1.433* (1.195, 1.720)	1.386* (1.155, 1.662)	1.352* (1.125, 1.624)
***Household-level variables***			
Household wealth quintile (ref: Very Poor)			
Quintile 2	0.871*** (0.750, 1.012)	–	0.869*** (0.746, 1.012)
Quintile 3	0.839** (0.709, 0.993)	–	0.850*** (0.716, 1.008)
Quintile 4	0.886 (0.735, 1.066)	–	0.888 (0.736, 1.072)
Quintile 5(Very Rich)	0.901 (0.732, 1.109)	–	0.890 (0.723, 1.096)
Gender X Quintile (ref: Poor and very poor)			
Male X Quintile 3	–	0.802** (0.682, 0.943)	–
Male X Quintile 4	–	0.800** (0.670, 0.956)	–
Male X Quintile 5	–	0.673* (0.551, 0.822)	–
Female X Quintile 3	–	1.516* (1.154, 1.992)	–
Female X Quintile 4	–	1.857* (1.430, 2.412)	–
Female X Quintile 5		2.597* (1.983, 3.402)	–
Religion (ref: Islam)			
Orthodox	0.962 (0.800, 1.157)	0.945 (0.787, 1.134)	0.934 (0.778, 1.121)
Catholic	0.962 (0.775, 1.194)	0.948 (0.765, 1.174)	0.945 (0.762, 1.173)
Bektashi	0.637** (0.435, 0.933)	0.631** (0.432, 0.922)	0.647** (0.443, 0.947)
Atheist	1.502*** (0.981, 2.300)	1.477*** (0.971, 2.246)	1.492*** (0.982, 2.268)
Other	1.009 (0.500, 2.035)	1.014 (0.508, 2.024)	1.046 (0.522, 2.098)
***Community-level variables***			
Urban (ref: Rural)	1.460* (1.275, 1.671)	1.430* (1.249, 1.636)	1.451* (1.267, 1.662)
Regions (ref: Tiranë)			
Berat	0.675* (0.525, 0.867)	0.699** (0.544, 0.897)	0.698** (0.543, 0.898)
Dibër	0.770** (0.618, 0.958)	0.797** (0.641, 0.991)	0.776** (0.623, 0.966)
Durrës.	0.581* (0.463, 0.729)	0.577* (0.461, 0.723)	0.582* (0.464, 0.731)
Elbasan	0.708* (0.579, 0.867)	0.731* (0.598, 0.893)	0.721* (0.589, 0.882)
Fier	0.512* (0.410, 0.639)	0.527* (0.423, 0.657)	0.529* (0.424, 0.660)
Gjirokastër.	0.758** (0.581, 0.990)	0.772*** (0.593, 1.006)	0.765** (0.587, 0.997)
Korçë	0.621* (0.499, 0.772)	0.633* (0.510, 0.787)	0.639* (0.514, 0.794)
Kukës	0.506* (0.404, 0.634)	0.514* (0.411, 0.643)	0.505* (0.403, 0.632)
Lezhë	0.432* (0.319, 0.585)	0.449* (0.332, 0.608)	0.444* (0.327, 0.601)
Shkodër.	0.687* (0.568, 0.881)	0.700** (0.548, 0.896)	0.692* (0.540, 0.885)
Vlorë	0.944 (0.747, 1.192)	0.965 (0.767, 1.215)	0.962 (0.763, 1.213)
β_ehat_	2.915* (2.325, 3.655)	2.767* (2.206, 3.469)	2.728* (2.175, 3.422)
Constant	0.031* (0.021, 0.048)	0.017* (0.011, 0.026)	0.0523* (0.034, 0.081)
***Random part***			
ρ^a^	0.044	0.042	0.042
ψ^b^	0.150 (0.101, 0.223)	0.144 (0.096, 0.216)	0.145 (0.097, 0.217)
LR test statistic^c^	43.59*	40.48*	41.54*
Level 1 Units (N)	25,111	25, 111	25,111
Level 2 Units	715	715	715

*1% significance level, ** 5% significance level, ***10% Significance level

^a^ Intra-cluster correlation

^b^ Variance of the random-intercept term

^c^ Comparing random-intercept logistic model against ordinary logit model

The results of Model 1, Table 3, show that all explanatory variables had the expected signs and most were statistically significant. The time dummy estimates indicate that the probability of smoking decreased by 23% in 2017–18 compared to 2008–09. The inclusion of a time dummy allowed us to capture any changes over time in smoking behavior, including prices of cigarettes. To understand the separate effect of prices on the likelihood of smoking, we also ran a separate regression, as a robustness check, by including tax-inclusive retail prices (in international dollars PPP) for 2008 and 2018 for the most sold brand of cigarettes (for a pack of 20 cigarettes), as reported in the WHO Global tobacco epidemic report [22]. To avoid the multicollinearity problem, this estimation included the retail price variable instead of the time dummy. The results of the price variable indicate that a 10% increase in prices decreases the likelihood of smoking by 1.2%, while the direction and magnitude of the results of other variables remained the same as those in the time dummy specification.

Male respondents are 12 times more likely to smoke than females. Compared to those with university education (reference category), individuals with no or less than 4-years of primary education were 1.5 times more likely to smoke. Adults in the age group 25–44 are 3.2 times more likely to smoke than the younger age groups (15–19).^9^ Similarly, divorced individuals and skilled manual workers were more likely to smoke compared to their counterparts. Compared to moderate drinkers (reference category), binge drinkers are 59% more likely to smoke, while non-drinkers are 58% less likely to smoke. Individuals with health awareness are 46% less likely to smoke.

The likelihood of smoking decreased with increasing household wealth, though results are only significant for poor and middle-income quintiles, a somewhat surprising result as the descriptive statistics showed that smoking prevalence among these groups is lower than for richer groups. Urban dwellers are 46% more likely to smoke than rural residents. While we found significant regional variations in tobacco smoking, residents of all other prefectures are less likely to smoke than Tiranë residents. Specifically, residents from Lezhë are 57% less likely to smoke than Tiranë residents followed by residents of Fier and Kukës (49%), respectively.

Results of Model 2, Table 3, show that all estimates maintain the same qualitative effect of the independent variables, with negligible change in the odds ratios. The following few points are worth noting. First, the likelihood of smoking decreases by income quintiles among males. Males from the richest quintiles are 33% less likely to smoke than males from the bottom two quintiles. Second, the likelihood of smoking increases by income quintiles among females, with the richest females being almost 1.6 times more likely to smoke tobacco than poor females (bottom two quintiles). Third, with the inclusion of the quintile by gender interaction term, the main effect of the likelihood of smoking for males increases. Males, in model 2, are almost 25 times (up from 12 times in Model 1) more likely to smoke than females. One possible explanation is that in our sample we noticed different patterns of distribution of male/female smoking by wealth. Males who smoke are almost uniformly distributed among wealth quintiles, while the distribution of females who smoke is skewed toward the very rich quintile. More specifically, 36.66% of males who smoke are very poor and poor (the reference category) compared to 17.28% of females who smoke. In contrast, 44.36% of females who smoke are very rich compared to 21.2% of males. Fourth, while in Model 1, the rich quintiles had no statistically significant effect on the likelihood of smoking, the interaction of wealth with gender (Model 2) yielded statistically significant results for all quintiles.

Results of Model 3, Table 3, show that inclusion of gender and education interaction terms produces a negligible change in the odds ratios. The following points are worth noting. First, consistent with descriptive statistics, the likelihood of tobacco smoking decreases with higher education among males. Males with no education are (2 times) more likely to smoke tobacco than university educated males. Second, similar to results on males, females with no education are 75% more likely to smoke than university educated females. However, this pattern is reversed for a higher level of education where females with primary or secondary education are less likely to smoke than university educated females, by 48 and 40%, respectively. Third, with the inclusion of education by gender interaction terms, the males are only 4 times (decrease from 12 times in Model 1) more likely to smoke than females. This could be due to the different patterns of distribution of male/female smoking by education level. A closer examination of the data reveals that 41.8 and 41.6% of male smokers have primary and secondary education, respectively, compared to about 24% of female smokers being in each of these educational levels. In addition, 14% of male smokers have completed university education (the reference category) compared to 46.3% of female smokers.

Table 4 reports the results on the frequency of smoking. The likelihood ratio test favors the negative binomial model over the Poisson model. While the direction of coefficients in the frequency model remains similar to tobacco use, many variables were statistically insignificant.

**Table 4:** Regression results for frequency of smoking (*N* = 3277)

Variables	Coefficient (95% CI)
***Individual-level variables***	
Survey year (ref: 2008–09)	−0.021 (−0.060, −0.018)
Gender (ref:male)	0.623* (0.570, 0.676)
Age (ref: 15–19)	
20–24	0.248* (0.158, 0.338)
25–44	0.276* (0.189, 0.362)
45–49	0.257* (0.161, 0.354)
Education (ref: University and Post-graduate)	
No education/Primary less than 4-year	0.217* (0.099, 0.336)
Primary 8-year	0.147* (0.085, 0.209)
Secondary/Professional/technical	0.114* (0.058, 0.171)
Alcohol consumption (ref: Moderate Drinker)	
Non drinker	−0.005 (−0.047, 0.037)
Binge drinker	0.190* (0.079, 0.301)
Health awareness on Smoking	0.006 (−0.109, 0.122)
Marital Status (ref: Not married)	
Married	0.026 (−0.021, 0.073)
Divorced	0.140** (0.031, 0.248)
Occupation (ref: Unemployed)	
Professional/Clerical/Sales/Services	0.021 (−0.032, 0.073)
Skilled Manual	0.028 (−0.025, 0.081)
Agriculture	−0.002 (− 0.071, 0.301)
Unskilled Manual/Other	−0.015 (− 0.082, 0.053)
***Household-level variables***	
Household wealth quintile (ref: Very Poor)	
Quintile 2	−0.001 (− 0.058, 0.056)
Quintile 3	−0.003 (− 0.067, 0.061)
Quintile 4	− 0.015 (− 0.085, 0.054)
Quintile 5(Very Rich)	−0.003 (− 0.081, 0.075)
Religion (ref: Islam)	
Orthodox	0.021 (−0.050, 0.093)
Catholic	−0.020 (− 0.097, 0.058)
Bektashi	−0.040 (− 0.189, 0.109)
Atheist	0.095 (0.056, 0.245)
Other	−0.173 (− 0.479, 0.132)
**Community-level variables**	
Urban (ref: Rural)	0.069** (0.020, 0.118)
Regions (ref: Tiranë)	
Berat	−0.131* (− 0.214, − 0.048)
Dibër	0.003 (− 0.071, 0.075)
Durrës	0.024 (− 0.055, 0.102)
Elbasan	−0.083** (− 0.150, − 0.017)
Fier	−0.171* (− 0.248, − 0.095)
Gjirokastër.	−0.182* (− 0.276, − 0.088)
Korçë	−0.055 (− 0.134, 0.023)
Kukës	− 0.048 (− 0.125, − 0.030)
Lezhë	0.159* (0.054, 0.264)
Shkodër.	− 0.016 (− 0.100, 0.068)
Vlorë	−0.047 (− 0.124, 0.030)
β_ehat_	0.147* (0.058, 0.236)
LR test statistic^a^	3912.75*
lnalpha	−1.674 (−1.744, −1.603)

*1% significance level, ** 5% significance level, ***10% Significance level

^a^ Likelihood ratio test of alpha = 0

## Discussion

Our findings on urban residents, Tiranë residents, and 25–44 old-aged participants being more likely to smoke tobacco than their counterparts are consistent with others [5, 15–17]. We also find that skilled manual workers were more likely to smoke than other occupations, which could be explained by the degree of enforcement for smoke-free regulation. Although Albania’s smoke-free law applies to both public and private workplaces, WHO (2019) reports an overall compliance score of 5 out of 10, with indoor offices and workplaces having a low score of 5 [6]. Assuming that many skilled manual workers have workplace environments that make enforcement challenging, they will have more opportunity to smoke and sustain their behavior. Also, given the considerable size of the construction sector, one could expect the manual, skilled construction workers to primarily work outdoors, thus having more opportunity to smoke.

Additionally, one possible common explanation for all these findings is the high concentration of economic activity in urban centers, particularly in Tiranë, which affords these groups a higher purchasing power and higher consumption of tobacco products.^10^ Given that the Tobacco Control policy is national in scope, our finding about the regional variation in tobacco smoking raises the question of whether the regional variation is related to various degrees of enforceability of the policy or the accessibility of tobacco products across regions, a question which requires further investigation.

Similar to Toçi et al. (2014) and Pipero et al. (2015) findings about Albanian youth, we also find that tobacco smoking is linked to alcohol consumption [15, 16]. Unlike these studies, our findings apply to adult population for whom the risk of sustaining practices of drinking and smoking is much more relevant than the youth’s risk of initiating such practices. We also use the 2SRI estimation method to address the mutual feedback of these behaviors and obtain unbiased estimates. Our more specific finding that binge drinkers are 59% more likely to smoke tobacco than moderate drinkers suggest that these risky health behaviours that feed on each other should be taken seriously. They should be considered as the addicting behaviours with serious adverse health and social effects, and not as the beneficial socializing practice for human connectedness, a more commonly held view in the Albanian culture. Additionally, smoking cessation attempts for many individuals could be impaired by their drinking habits and addressing these behaviors jointly may be needed for those individuals who engage in both smoking and intense alcohol consumption.

Our findings that the likelihood of smoking decreased by 23% in 2017–18 compared to 2008–09 suggests that the tobacco control efforts have been a smoking deterrence. It is important to interpret this result with caution as our data is cross-sectional and to properly measure the impact of Tobacco Control policy, utilization of longitudinal data is needed.

Consistent with others [5, 7, 15–17], we find that male respondents are considerably more likely to smoke than female respondents. This pattern reflects the traditionally high, social acceptability of smoking among men. While smoking among females was typically frowned upon during communism, it slowly became more socially acceptable since younger generations view smoking as a sign of female emancipation [7]. Moreover, at the onset of the transition period, in the absence of any Tobacco Control policy in post-communist countries, the tobacco industry’s skillful and successful marketing efforts were particularly aggressive towards female smokers [4]. Often these marketing strategies portrayed smoking as a symbol of empowerment, glamour, emancipation, and success and contributed to increased female smoking prevalence in these countries, Albania included [3, 4]. As noted earlier, this rising prevalence of female smoking is troubling, and our extended models provide some valuable insights on gender smoking behavior in Albania.

Consistent with smoking literature [24], the likelihood of smoking decreases with wealth among Albanian males. There is, however, a contrasting pattern for females, namely smoking increases with wealth. This contrast leads to some interesting policy implications in targeting the groups that are most likely to smoke, namely poor males and rich females.

While there are concerns that most economically vulnerable groups are at higher health risk, the low-income population will likely respond more to price-based incentives like taxation, due to the higher income effect of the price increase. The tobacco tax has increased over time [13]. Since 2014, the tax portion of cigarette prices is about 67%, which includes an excise rate of 49%. Were Albania to increase the excise rate to 75%, as per WHO FCTC minimum rate recommendation, the projected smoking reduction is 24.5% within 15 years [11]. Empirical work for many countries has consistently shown that taxation is the most effective instrument in reducing smoking [25, 26]. Our finding that poor males are more likely to smoke than rich males also supports the notion that a much higher excise tax rate than the current 49% could be very effective in deterring smoking, particularly among poor males.

Another implication of our finding is that although rich females may not respond to increased taxation to a similar degree as the poor males, having the financial means, they could instead be much more responsive to take on cessation. Along with raising taxes, WHO’s MPOWER (2008) policy package recommends smoking cessation and warning interventions [27]. These include: communication campaigns promoting health risks of smoking; increasing the availability and accessibility of nicotine-replacement therapy (NRT) products such as gum, nasal spray, patch, lozenge, oral inhaler; quit/help line programs that offer advice and support to quitters; and capacity building within the health system to educate and train health professionals to deliver smoking cessation and prevention programs [27]. These cessation interventions should be gender-based since women face unique barriers in their attempts to quit smoking. More specifically, women are faced with physiological factors such as fear of weight gain, or fear of not managing mood changes due to the effects of hormonal and menstrual cycles [3]. It is also understood that women are more prone to the risk of depression and may need more social support when attempting to quit [3].

WHO (2019) reports that Albania has a moderate cessation policy that demands more attention and further development [6]. For instance, cessation support services are only offered in some Albanian health clinics or other primary care facilities. However, there are no toll-free help-lines in place to support those attempting cessation. There are also no NRT products available in the market, and their costs are not generally covered by insurance [6]. We suggest that a comprehensive tobacco cessation policy, specifically targeting rich females, could successfully be implemented in Albania. The more affluent female smokers are better positioned to partake on NRT therapies and perhaps absorb the cost, as an out-of-pocket expense. Since the private market has not facilitated the introduction of these NRT therapies, the government could play a role in facilitating the availability and accessibility of these products.

Successful cessation programs from other European countries such as Sweden and Romania indicate that women are more inclined than men to utilize the quit-lines services [3]. In other programs, smoking cessation was promoted to women during medical treatment for unrelated conditions for which smoking was deemed to be a health risk. In Spain for instance, while promoting neonatal health, pregnant women were proactively encouraged to quit smoking during pregnancy. In Italy, cervical cancer screening was used as an opportunity to encourage cessation [3].

While these examples may not be fully applicable in the Albanian context, they suggest that such nudges from healthcare providers are often important catalysts for people to quit. Given the common understanding that high-income and better educated individuals are more proactive in utilizing care [28], cessation approaches to rich and educated females could be promoted as they interact with the healthcare system. Ideally, these cessation measures would benefit all women regardless of their socioeconomic status. Overall, successful cessation programs will require a strong commitment and concerted efforts on part of the Albanian government in partnership with healthcare providers, non-governmental organizations, including women’s groups and community organizations.

Consistent with the existing studies [29, 30], our Model 1 and 2 findings show that the likelihood of tobacco smoking decreases with higher education. With the education and gender interaction in Model 3, this pattern only holds for males, while female smoking behaviour is more involved. While females with no education are 75% more likely to smoke, females with primary or secondary education are less likely to smoke than university educated females. Although paradoxical at first glance, the latter is partially explained by the prevailing mindset that smoking is a signal of female emancipation. The tobacco industry, in the absence of effective control regulations, targeted girls and women in many post-communist countries, by presenting an image linking smoking with sophistication, independence, and freedom [3].

Given that better educated individuals are likely to be more responsive to educational and awareness campaigns about the health hazards of smoking, targeting more educated females in such campaigns with messages that decouple smoking from the notion of female liberation could be a worthy effort. Our finding that the likelihood of smoking decreased by around 46% if individuals are aware of the adverse health effects of smoking, reinforces the policy importance of continuously undertaking awareness campaigns to reduce smoking.

Unfortunately, in Albania, there were no anti-tobacco mass media campaigns aired during 2016 and 2018 periods [6]. WHO Europe (2016), estimates that the impact of more intense mass media campaign in reducing smoking prevalence is the second highest (6.3% reduction within 15 years) after taxation [11]. This highlight the importance of undertaking awareness campaigns in the future. These campaigns could be run in partnership with healthcare providers, community organizations that engage youth and women, and integrated into school curriculums through the form of health education**.** In particular, anti-tobacco mass media campaigns (such as through radio, TV, cell text messages, or social media), have the potential to not only create awareness about the harms of tobacco use and second-hand smoke but also encourage quitting [3, 27].

Lastly, some women-specific interventions proposed above may apply beyond Albania and to other countries that are experiencing similar patterns of female smoking behavior within similar socio-economic contexts. For instance, educational and awareness campaigns on the harmful effects of smoking that also deliberately attempt to neutralize the notion of smoking as a signal of female emancipation could universally apply to other post-communist countries. While acknowledging the benefits of other programs, we cautionary suggest that their design is first and foremost informed by the understanding of the smoking behavior of the demographic group of interest in order to achieve the desired response.

The following limitations of this study are worth noting. First, the data on tobacco smoking may be subject to recall bias as it was self-reported. Second, the pooled survey data used in this study does not allow us to track the smoking behavior of individuals over time and hence results on time dummy should be interpreted with some caution. Third, the DHS wealth index has been criticized for being too urban in its construction [31]. Fourth, given the different social contexts of post-communist countries, our findings cannot be widely generalized to other transitional countries of Eastern Europe that are similarly experiencing an increase in female smoking prevalence. Nevertheless, our study provides a useful framework to understand female smoking behavior and hence target various policy units appropriately. Finally, we acknowledge that evaluating the effectiveness of the tobacco control policies in Albania is an important issue to examine, but data limitations preclude us from undertaking such a task in this paper. The Tobacco Control Score developed by Joossens and Raw (2006) is a useful methodology for assessing different policy measures [32]. Since the scores are based on expert opinion, generating the data would require collaboration with the Albanian tobacco control experts. Nevertheless, the empirical evaluation of the effectiveness of tobacco control policy in Albania is an important avenue for future research.

## Conclusion

Using 2008–09 and 2017–18 ADHS and a random intercept logistic model with a two-stage residual inclusion estimation method, this study examines the influence of various socioeconomic, demographic, and behavioral factors on the likelihood and frequency of tobacco smoking in Albania. Our findings suggest that while overall smoking prevalence has declined overtime, more efforts are needed to contain this epidemic. The current Albanian Tobacco Control policy, if enforced properly and combines price-based incentives like taxation with cessation approaches and educational awareness campaigns, will continue to deter smoking. Our findings of significant inter-quintile and inter-educational differences in smoking practices within each gender category further suggest that targeting various demographic groups in differentiated fashion (based on the understanding of their specific smoking behavior) within the existing tobacco control framework could further enhance its effectiveness.

## Data Availability

This study used a dataset available from USAID’s The DHS Program and can be accessible at https://dhsprogram.com/data/available-datasets.cfm

## Abbreviations

ADHS: Albanian Demographic and Health Survey
DHS: Demographic and Health Surveys
FCTC: Framework Convention on Tobacco control
WHO: World Health Organization
2SRI: Two-stage residual inclusion

## Footnotes

1 Detailed information on survey methodology can be found in the Institute of Statistics, 2010, 2018 [8, 10].

2 The analysis is restricted to age group 15–49 as the DHS did not collect data for aged 50–59 in the 2008 survey year.

3 The use of tobacco smoking, other than cigarettes, constitutes less than 1% of pooled responses.

4 In 2017–18, respondents were asked: “Do you believe that smoking causes serious health problems, minor health problems or no health problems at all?” In 2008–09, respondents were asked: “In what ways do you believe smoking can cause health problems such as lung or throat cancer, asthma, heart disease, stroke, complications in pregnancy, etc.”

5 DHS calculates the wealth index based on a standard set of household assets, dwelling characteristics such as the source of drinking water, toilet facilities, and flooring materials, and ownership of consumer items ranging from a television to a bicycle or car.

6 We did not control for unobserved community-level factors in modeling the frequency of smoking due to the low value of intra-cluster correlation.

7 As mean differences in the prevalence rate over the two survey years were statistically insignificant within education and income quintiles in most of the cases, we decided to conduct the pooled cross-section analysis which includes a time dummy. This time dummy allows us to capture time effects in tobacco smoking between the two survey years.

8 Only 3.36% (*N* = 875) of the total sample (*N* = 25,986) were excluded due to missing information on alcohol consumption (376) and health awareness (499).

9 Testing for the equality of coefficients on age 25–29, 30–34, 35–39, and 40–44 age groups suggest that they could be aggregated.

10 Tiranë is the most populous urban center in Albania and also accounts for the highest economic activity (as measured by GDP). In 2017, the per capita GDP of the Tiranë district was almost 40% higher than the national average [23]. Additionally, the pooled DHS data show that Tiranë has the highest concentration of manual skilled labor, with 21% of workers in this category residing in Tiranë and has the second highest smoking rate in the country.
